# Editorial: Dynamical Networks of Life/Death Decisions in a Cell: From DNA Repair to Cell Death

**DOI:** 10.3389/fcell.2021.722426

**Published:** 2021-07-16

**Authors:** Inna N. Lavrik

**Affiliations:** Translational Inflammation Research, Medical Faculty, Center of Dynamic Systems, Otto von Guericke University Magdeburg, Magdeburg, Germany

**Keywords:** cell death, DNA damage, DNA repair, mitophagy, BER

Life/death decisions in the cell are controlled by the intricate balance between cell death and survival signaling pathways. DNA damage induces multifactorial cellular responses: from DNA repair to cell death. In the last years, there has been emerging evidence indicating regulatory crosstalk between these cellular networks. It turned out that core components of cell death networks control DNA repair as well as key regulators of the DNA repair machinery play a major role in the cell death (Boege et al., 2017; Alemasova and Lavrik, 2019; Muller et al., 2020). The crosstalk between these networks is rather complex due to several pathways of DNA repair as well as more than a dozen types of programmed cell death that can be initiated upon DNA damage: apoptosis, necroptosis, autophagy, ferroptosis and paranathosis (Galluzzi et al., 2018). Getting new insights into these intricate machineries and their crosstalk is addressed in this collection. This research is highly important for new directions of development in contemporary biomedicine and paves the way towards drugs development involving targeting cell death and DNA repair networks.

The major part of the collection features the molecular mechanisms of DNA repair, in particular, focusing on base excision repair (BER). A comprehensive overview of the BER pathway in the model organism, *Caenorhabditis elegans* (*C. elegans*) is given by Elsakrmy et al. highlighting BER among other pathways of DNA repair. Another review by Bayken et al. presents state of the art knowledge on the role of BER in the processing of complex DNA structures, which are triggered by oxidative stress and anticancer drugs. Uncovering the structural features of histone-DNA crosslinks and molecular mechanisms of their repair is the subject of the review: by Pachva et al. The latter knowledge plays a key role in the development of novel targeted approaches associated to the induction of DNA repair pathways and DNA damage-induced cell death.

Valuable insights into the molecular mechanisms of the BER machinery are provided by the research articles in this collection. In particular, a detailed analysis of substrate specificity of several key enzymes of the BER pathway is carried out using *in vitro* biochemical systems. The comparison of substrate specificity of human apurinic/apyrimidinic (AP) endonuclease APE1 toward DNA and RNA substrates has allowed finding out new features of APE1 specificity toward DNA and RNA of non-canonical structure (Davletgildeeva et al.). The influence of human tyrosyl-DNA phosphodiesterase 1 (TDP1) and APE1 on the molecular architecture of the AP1 site has been uncovered by Lebedeva et al. This analysis revealed the role of these two enzymes in the crosslinking of 8-oxoguanine-DNA glycosylase (OGG1) to the APE1 site as well as dissected the interplay of OGG1, PARP1, and PARP2 at the initial stages of the BER pathway. The activity of PARP1 and PARP2 in *Arabidopsis thaliana* was compared by Taipakova et al., which allowed unraveling a new type of DNA-modifying activity. Furthermore, a new type of substrate activity of tyrosyl-DNA phosphodiesterase 1 (TDP1) was found by Dyrkheeva et al. Moreover, a comparison of the activity on DNA substrates of endonuclease VIII-like 1 (NEIL1), human 8-oxoguanine-DNA glycosylase (OGG1), endonuclease III (NTH1), prokaryotic formamidopyrimidine-DNA glycosylase (Fpg), and endonuclease VIII (Nei) has been carried out by Kuznetsova et al. These studies identify new distinct and common features of these enzymes, which further underlines the high complexity of the BER machinery and the importance of its investigation in different organisms.

New insights into the other DNA repair pathway, nucleotide excision repair (NER), have been obtained by Petruseva et al. focusing on uncovering the patterns of XPD-p44 interactions with bulky DNA damages. The molecular mechanisms of non-homologous end joining (NHEJ) pathway in cancer cells upon ionizing radiation (IR) are analyzed by Tumia et al. In particular, it has been demonstrated that IR leads to downmodulation of NHEJ repair processes by inhibiting the synthesis of NHEJ repair proteins including Ku70, Ku80, and DNA-PKcs. These findings reveal the molecular basis of the NHEJ repair machinery in cellular responses to drug/radiation-induced DNA damage and development of anti-cancer drugs.

Studies of the autophagic pathway, mitochondrial respiration and cross-talk of autophagy with different forms of cell death also take a prominent place in this collection. The crosstalk of different forms of cell death focusing on the balance between mitophagy and apoptosis is analyzed by Wohlfromm et al. In this study, it was uncovered that the intricate balance between these two pathways might both inhibit and block apoptosis at the earlier and later time points, respectively. The role of HDAC6 in Transactive response DNA-binding protein 43 (TDP-43)-induced neurotoxicity and contribution of autophagy to this pathway and to the development of amyotrophic lateral sclerosis (ALS) is analyzed by Lee et al. The effects of mTOR inhibitors, rapamycin or torin1, on the germination of wheat seeds is analyzed by Smailov et al. Finally, the role of E3 Ubiquitin Ligase Hyd activity in the balance between mitosis and cell death during *Drosophila* oogenesis, is studied by Dorogova et al.

Taken together, this collection provides new insights into life/death decisions in the dynamical networks of DNA damage and cell death, identifies new targets in this network as well as paves the way toward novel therapeutic applications.

## Author Contributions

The author confirms being the sole contributor of this work and has approved it for publication.

## Conflict of Interest

The author declares that the research was conducted in the absence of any commercial or financial relationships that could be construed as a potential conflict of interest.

## References

[B1] Alemasova E. E. Lavrik O. I. (2019). Poly(ADP-ribosyl)ation by PARP1: reaction mechanism and regulatory proteins. Nucleic Acids Res. 47, 3811–3827. 10.1093/nar/gkz120 30799503 PMC6486540

[B2] Boege Y. Malehmir M. Healy M. E. Bettermann K. Lorentzen A. Vucur M.. (2017). A dual role of caspase-8 in triggering and sensing proliferation-associated DNA damage, a key determinant of liver cancer development. Cancer Cell 32, 342–359 e10. 10.1016/j.ccell.2017.08.010 28898696 PMC5598544

[B3] Galluzzi L. Vitale I. Aaronson S. A. Abrams J. M. Adam D. Agostinis P.. (2018). Molecular mechanisms of cell death: recommendations of the Nomenclature Committee on Cell Death 2018. Cell Death Differ. 25, 486–541. 10.1038/s41418-017-0012-4 29362479 PMC5864239

[B4] Muller I. Strozyk E. Schindler S. Beissert S. Oo H. Z. Sauter T.. (2020). Cancer cells employ nuclear caspase-8 to overcome the p53-dependent G2/M checkpoint through cleavage of USP28. Mol. Cell. 77, 970–984 e7. 10.1016/j.molcel.2019.12.023 31982308 PMC7060810

